# Defining and Risk-Stratifying Immunosuppression (the DESTINIES Study): Protocol for an Electronic Delphi Study

**DOI:** 10.2196/56271

**Published:** 2024-06-06

**Authors:** Meredith Leston, José Ordóñez-Mena, Mark Joy, Simon de Lusignan, Richard Hobbs, Iain McInnes, Lennard Lee

**Affiliations:** 1 Nuffield Department of Primary Care Health Sciences University of Oxford Oxford United Kingdom; 2 Wolfson Medical School Building University of Glasgow Glasgow United Kingdom; 3 Department of Oncology University of Oxford Oxford United Kingdom

**Keywords:** immunosuppressed, immunocompromised, COVID, vaccines, COVID-19, surveillance, phenotype, adult, immunosuppression, clinical risk, disease surveillance, clinical consensus, eDelphi, immunosuppressed patient, immunosuppressed patients, study design, Delphi, methods, methodology, statistic, statistics, statistical, consensus, immune, immunity, immunology, immunological

## Abstract

**Background:**

Globally, there are marked inconsistencies in how immunosuppression is characterized and subdivided into clinical risk groups. This is detrimental to the precision and comparability of disease surveillance efforts—which has negative implications for the care of those who are immunosuppressed and their health outcomes. This was particularly apparent during the COVID-19 pandemic; despite collective motivation to protect these patients, conflicting clinical definitions created international rifts in how those who were immunosuppressed were monitored and managed during this period. We propose that international clinical consensus be built around the conditions that lead to immunosuppression and their gradations of severity concerning COVID-19. Such information can then be formalized into a digital phenotype to enhance disease surveillance and provide much-needed intelligence on risk-prioritizing these patients.

**Objective:**

We aim to demonstrate how electronic Delphi objectives, methodology, and statistical approaches will help address this lack of consensus internationally and deliver a COVID-19 risk-stratified phenotype for “adult immunosuppression.”

**Methods:**

Leveraging existing evidence for heterogeneous COVID-19 outcomes in adults who are immunosuppressed, this work will recruit over 50 world-leading clinical, research, or policy experts in the area of immunology or clinical risk prioritization. After 2 rounds of clinical consensus building and 1 round of concluding debate, these panelists will confirm the medical conditions that should be classed as immunosuppressed and their differential vulnerability to COVID-19. Consensus statements on the time and dose dependencies of these risks will also be presented. This work will be conducted iteratively, with opportunities for panelists to ask clarifying questions between rounds and provide ongoing feedback to improve questionnaire items. Statistical analysis will focus on levels of agreement between responses.

**Results:**

This protocol outlines a robust method for improving consensus on the definition and meaningful subdivision of adult immunosuppression concerning COVID-19. Panelist recruitment took place between April and May of 2024; the target set for over 50 panelists was achieved. The study launched at the end of May and data collection is projected to end in July 2024.

**Conclusions:**

This protocol, if fully implemented, will deliver a universally acceptable, clinically relevant, and electronic health record–compatible phenotype for adult immunosuppression. As well as having immediate value for COVID-19 resource prioritization, this exercise and its output hold prospective value for clinical decision-making across all diseases that disproportionately affect those who are immunosuppressed.

**International Registered Report Identifier (IRRID):**

PRR1-10.2196/56271

## Introduction

At present, there is no clinical consensus around the conditions and medications that confer immunosuppressed status upon an individual [1]. This population scales from 2% [2] to over 10% [3] of the general population dependent on the definition applied, most notably when individuals with diabetes, malnourishment, or older age are incorporated [3]. This disagreement on what constitutes immunosuppression extends to how best to subdivide this heterogeneous population: out of the binary [4], continuum [5], and hierarchical [6] approaches available, there is currently no gold standard [7]. This inconsistency undermines ambitions for targeted care and disease surveillance as aggregate-level analysis dominates, and subtrends lose visibility [7].

Despite this, it is well known that patients who are immunosuppressed experience worse infection outcomes [8] and, in some cases, respond poorly to vaccination [9]. Meanwhile, there has been no concerted effort to differentiate vaccine side effects within those who are immunosuppressed [10]. Without a clinically meaningful and electronic health record–compatible means of identifying and subdividing adults who are immunosuppressed, it will not be possible to improve this poor resolution in vaccine benefit-risk profiling in this population. Clinical guidelines around vaccine dosing, scheduling, and boosting as well as policies on the targeted distribution of antivirals or passive forms of immunization (eg, monoclonal antibodies and convalescent plasma) fare poorly as a result [7].

This study aims to obtain clinical consensus on a risk-stratified phenotype of adult “immunosuppression” to be implemented within UK health databases as standard. The use of COVID-19 as our reference condition is justified by pandemic gains to the immunosuppressed literature base [11]. While findings of differential vulnerability for COVID-19 among adults who are immunosuppressed may not be fully generalizable to alternative infections or even pediatric patients, the specificity of COVID-19 immunosuppressed literature—inclusive of infection outcomes among extremely rare or complex diseases—enables comparisons across all conditions and medications cited by the *UK Immunisation Against Infectious Disease* manual (Green Book [12], Multimedia Appendix 1).

## Methods

### Study Design

The objective of this protocol is to demonstrate how the electronic Delphi (eDelphi) [13] study design will be used to surface a definitive and risk-stratified phenotype for adult immunosuppression based on vulnerability to COVID-19. This process will see panelists seek to align on the conditions to be included in the said definition, their respective levels of risk for severe COVID-19 outcomes and key risk dependencies (eg, time treated, diagnosed, or in remissions and dosage of medication received). Consensus will be determined by whether over ≥75% of panelists agree on definition contents and risk relationships. A range of consensus statements will also be presented and evaluated by this same ≥75% consensus target. These statements will assess panelist agreement on the heterogeneity of patients who are immunosuppressed and their COVID-19 infection outcomes as well as the accuracy of the draft phenotypes (multilevel and high- vs low-risk categorized) that will be presented between eDelphi rounds. The >75% consensus level is not arbitrary but is based on the systematic review of Delphi consensus definitions by Diamond and colleagues [14]; here, across a random sample of 100 successful Delphi investigations, 75% was the median threshold to establish consensus.

The Delphi technique aims to build consensus on prespecified topics by soliciting the opinions, testimonies, or judgments of experts (Delphi panelists) with successive, anonymized questionnaires [13]. This method is especially valuable for generating insight and informing decision-making on complex, sensitive, emerging, or underresearched subject matters [15]. Its anonymized nature reduces demand characteristics or the influence of dominant personalities that can skew results in unblinded exercises [16]. Delphi questionnaires are improved upon by embedding opportunities for panelist feedback between consensus-building rounds. The amount and type of questions presented, as well as the time available to reach consensus, determines the number of rounds attempted in each study.

The eDelphi method hosts these investigations entirely online. This widens the pool for recruitment as geographical limitations are removed. Data management advantages and time and cost savings have also made the eDelphi method more attractive than in-person and paper-based alternatives [17].

Although neither Delphi nor eDelphi studies are supported by unambiguous methodological guidelines, the present protocol has cross-referenced Conducting and Reporting Delphi Studies guidance [18] and recommendations from systematic reviews into successful Delphi execution [14]. Its publication is intended to maximize study quality and transparency.

### Ethical Considerations

This study will be coordinated by the Clinical Informatics and Health Outcomes Research Group at the Nuffield Department of Primary Care Health Sciences, University of Oxford. Separate ethical approval was sought for this work but deemed unnecessary after review by both the University’s dedicated Research Governance Ethics and Assurance Team and the Joint Research Office Study Classification Group. It was determined that all activities fell under “pre-research,” “priority setting,” or “survey”; as such, these would not be subject to the Department of Health’s UK Policy Framework for Health and Social Care Research [19] and would not be subject to sponsorship or research ethics review. This decision was corroborated when cross-referenced with the Health Research Authority’s dedicated review algorithm [20], attendant leaflet (*Defining Research* [21]), and Health Care Quality Improvement Partnership *Guide for Clinical Audit, Research and Service Review* [22].

### Study Management

A steering group comprised of senior staff members, a primary investigator, statistical supervisors, external research collaborators, and a patient champion who is immunosuppressed will contribute to the design and implementation of this study protocol, provide ongoing advice while the study is active, and assist in the interpretation, write-up, and dissemination of study results.

**Figure 1:**
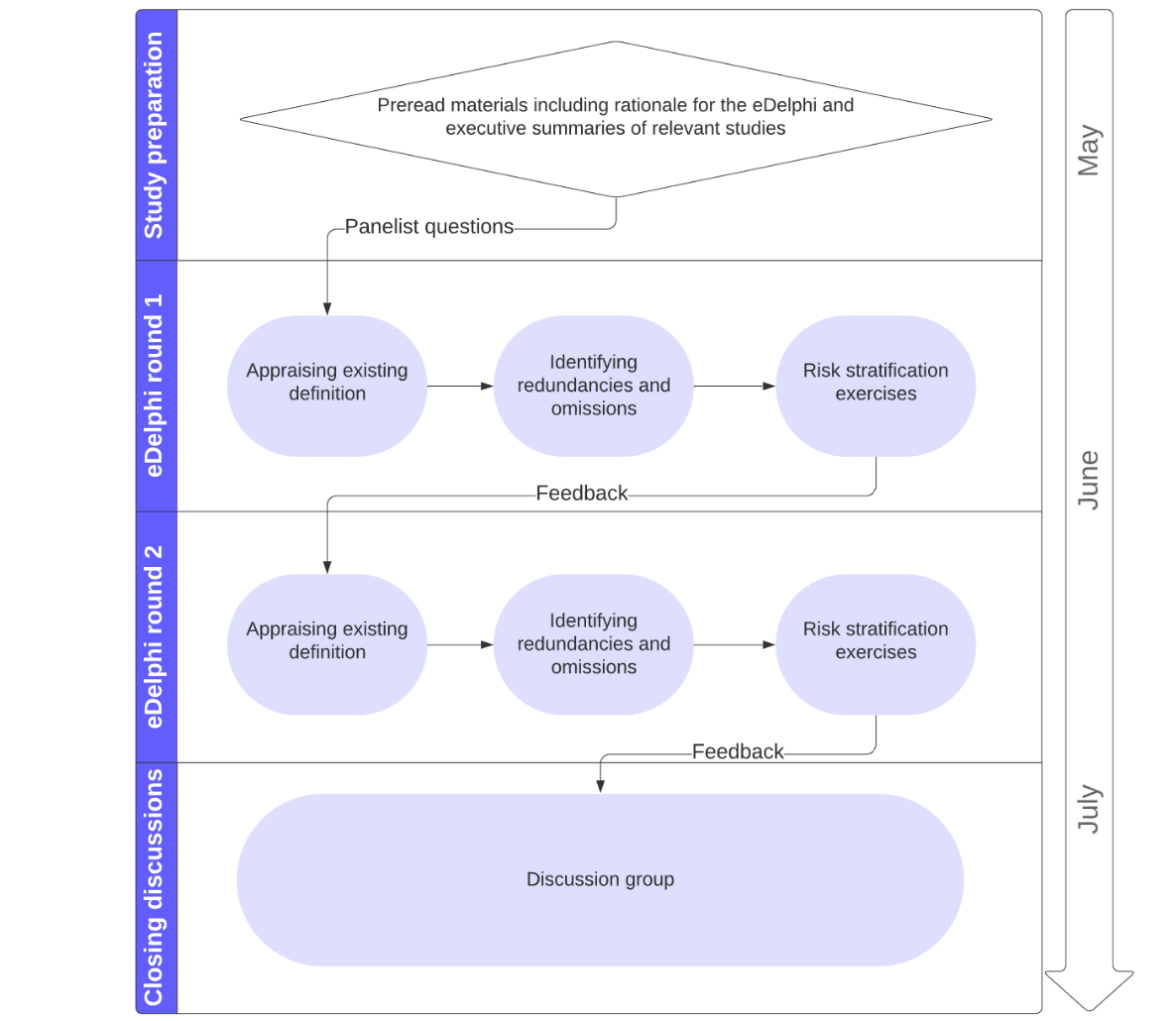
Schematic of the DESTINIES study timeline. DESTINIES: electronic Delphi Study to Define and Risk-Stratify Immunosuppression.

As illustrated in Figure 1, this study will involve a preparation period (to provide panelists with their consent form and preread materials), 2 rounds of consensus building, and a concluding discussion group to field final comments, points of clarification, confirmation, dissent, and feedback from panelists. Subject to successful recruitment, this study will run between May and July 2024. Panelists will be given 2 weeks to complete each round. To prevent attrition, reminders will be sent via email to nonresponders on days 6, 10, and 12. As per recruitment, telephone calls will be made on day 10 to encourage form submission; this will only occur when this information is listed publicly or has been provided by panelists who consent to it being used for this purpose.

### Recruitment

No sample size calculation is required for the eDelphi methodology; however, at least 50 specialists will make up the international eDelphi panel. This is intended to ensure that generalist and condition-specific experts are equally well-represented and to maximize the global footprint of this work. panelists will not be paid for their participation but are made aware that due attribution will be given to any outputs of this work should they be willing to be named.

Panelists will be recruited based on their affiliation with the following:

- World Health Organization Global Advisory Committee on Vaccine Safety
- Coalition for Epidemic Preparedness Innovations Scientific Advisory Committee
- The Global Immunocompromised Health Coalition
- The European Alliance of Associations for Rheumatology
- European Medicines Agency’s Vaccines Working Party
- Joint Committee on Vaccination and Immunization
- COVID-19 Neutralizing Monoclonal Antibodies (nMABs) and Antivirals Access Independent Advisory Group
- UK Scientific Advisory Group for Emergencies
- Independent Scientific Advisory Group for Emergencies
- Centers for Disease Control and Prevention Advisory Committee on Immunization Practices
- Food and Drug Administration Vaccine Advisory Panel
- The Nuffield Department of Primary Care Health Sciences
- The Nuffield Department of Medicine

Beyond this, inclusion will be dependent upon the credentials of prospective panelists—clinical, academic, or policymaking experience in vaccinology or immunology is essential. Willingness to use Google Forms to submit survey responses is desirable, but not essential. Paper-based versions of each eDelphi round will be provided for those either uncomfortable or unable to use this platform. Prospective panelists will be excluded, however, if they are unable to commit to the full study duration or their expertise is entirely pediatric.

Invitations to participate will be sent via email and managed by the primary investigator. When publically available, the primary investigator will follow up with invitations by telephone. The email invitation will outline the aims of this study, participation details, the level of commitment expected, and the inclusion and exclusion criteria specified. To maximize recruitment, those contacted will also have the opportunity to signpost figures in their network that adhere to the inclusion and exclusion specified.

Those who accept to participate will be allocated random identification numbers. Panelists will not be known to one another until the end of data collection. Preread materials will also be distributed. In this, panelists will be provided with a panelist information sheet, a brief rationale of this study, executive summaries of relevant steering group research outputs, and a consent form. Executive summaries include a systematic review of differential vulnerability to COVID-19 [23], a phenotyping methodology paper (publication imminent), and real-world evidence for differential vaccine response and COVID infection outcomes (observational cohort trial T cells, antibodies, and vaccine efficacy in SARS-CoV-2 [24] and investigation of COVID-19 risk among populations who are immunocompromised [25] studies, respectively). Consenting panelists will be asked to sign and return their consent forms to researchers and retain a copy for their records.

Panelists will be encouraged to ask investigators any clarifying questions on the preread materials or study design before the first round begins. The panelist information sheet will also be presented before each active eDelphi round to remind panelists of study objectives and their rights to withdraw.

Panelists who fail to respond to an eDelphi round after three consecutive email reminders will be defined as withdrawn. Panelists can also make their own requests to withdraw, however, data collected up to that point of participation cannot be erased. To assess potential attrition bias, the number, percentage, and characteristics of withdrawn panelists will be reported and compared to those who continue to participate. A withdrawal rate greater than one-third would be considered an unacceptable loss to follow-up. In this instance, this study would be discontinued and recruitment reopened.

### Questionnaire Design

The stated objectives of this study require that consensus be built around the definition of adult immunosuppression and digital phenotypes based on observed vulnerability to severe COVID-19 outcomes. This involves establishing and risk-stratifying constituent conditions and determining their dependencies (eg, time, dose).

To assess this, panelists will be presented with the complete list of conditions that, per the UK criteria for immunosuppression (Green Book chapter 14a [12], Multimedia Appendix 1), would lead to immunosuppressed status among adults. This resource has been selected on account of its expansiveness, its influence over vaccine allocation in the United Kingdom during this study’s period and its continuity with the systematic literature review included in preread materials. Leveraging their professional experiences and their understanding of preread findings, panelists will be asked to assess the appropriateness of each condition for inclusion in a definition of immunosuppression and to then evaluate their respective risk levels concerning COVID-19. The latter questions will be presented via Likert scale and binary “higher risk immunosuppressed” versus “lower risk immunosuppressed” options. Once this is completed, panelists will repeat this exercise for immunosuppressed conditions that are absent from the United Kingdom definition but are cited in comparable international resources (Immunisation Guidelines for Ireland [26], the Canadian Immunization Guide [27], the Australian Immunisation Handbook [28], the New Zealand Immunisation Handbook [29], US Yellow Book [30], US Pink Book [31] among others). Finally, consensus statements on how drug management, time since diagnosis or last treatment, duration of treatment, and duration of remission may modify vulnerability to COVID-19 will be presented via Likert agreement scale; these will be followed by more generalized consensus statements on the challenges associated with defining, treating, and protecting patients who are immunosuppressed from disease.

Collectively, these exercises will enable researchers to identify redundancies and omissions in the United Kingdom’s working criteria for immunosuppression and their respective vulnerabilities to severe COVID-19 outcomes; this data will then inform the construction of a risk-stratified phenotype of the patient spectrum that will be evaluated in the second eDelphi round and refined via the final discussion group. Although panelists will not be able to skip any questions presented, they will be able to indicate uncertainties in their answers. Optional feedback forms, again hosted on Google Forms, will be distributed via email between consensus-building rounds to clarify or refine questionnaire items if needed. Panelists will also be provided with a summary of results for each round to inform their subsequent responses and the concluding debate. Areas of agreement and disagreement will be discussed during the final discussion group.

### Data Protection

All study members will endeavor to protect eDelphi panel rights to privacy and informed consent, including adhering to the Data Protection Act, 1998 [32]. Each round will only collect the minimum required information for study purposes; panelists will not be known to each other. The primary investigator, however, will be required to know the panelists’ details for administrative purposes. Panelist data, including consent forms, completed surveys, and discussion audio files and transcripts, will be retained for 18 months before being destroyed.

eDelphi questionnaire rounds will be conducted on Google Forms. Google Forms’ functionalities include customizable questionnaire items, 1-time completion, advanced security measures (eg, data encryption, privacy protections, malware protections), real-time data insights, and automated Excel (Microsoft) spreadsheet generation and download. Paper-based copies of this questionnaire will be distributed to any panelist who declares discomfort using this platform, however. All panelists will be made aware of the importance of not sharing any sensitive or identifying information about patients in free text questionnaire items.

The final discussion groups will be hosted remotely on Zoom (Zoom Video Communications, Inc). Further, 3 discussion groups will be organized in total, breaking panelists into 3 groups based on time zone. Additionally, 4 time slots will be offered for each group. The time slot that receives the most votes will be taken forward. Panelists who are unable to attend any time slot offered will be connected to a Google Forms containing all items that will be discussed. This will ensure all panelists have been provided with the opportunity to contribute to final data collection. Panelists who can attend their final discussion group will be asked to turn their cameras off and not identify themselves by name at any point over the course of the discussion. Attendees will be reminded that discussions are recorded.

Only this study’s steering group will have access to study data. Computer-based information will be held securely and password-protected as per the standard. All data will be stored on a secure web server, Oxford Royal College of General Practitioners Clinical Informatics Digital Hub [33], hosted by the Nuffield Department of Primary Care Health Sciences. Access will be restricted by user identifiers, passwords, and multifactor authentication. Electronic data will be backed up every 24 hours to both local and remote media in an encrypted format.

Panelists will be offering their expertise within their capacity as a clinical, research, or policy professional. Panelists who consent to be named will be listed as coauthors in all study outputs; those who wish to remain anonymous will be acknowledged as part of the electronic Delphi Study to Define and Risk-Stratify Immunosuppression (DESTINIES) Consortium, where only professional affiliation will be listed. Study results will be made available to the public as well as relevant policy makers and academic institutions. Oversight from the European Alliance of Associations for Rheumatology People with Arthritis/Rheumatism across the Europe community will ensure that study results are available in a patient-accessible format.

## Results

Aggregated results of panelist response rate, level of agreement for each measure, and condition risk ranking will be calculated with R (version 4.3.1; Posit). As per Diamond and colleagues [14], consensus is reached when ≥75% of panelists agree on each item disputed. The analysis will be quality assured by the statistical supervision available within this study’s steering group. Areas of consensus and continued dissent will be content analyzed (inductive) and quantified and visualized via distributions of panel results. Panelist recruitment took place between April and May of 2024; the target set for over 50 panelists was achieved. The study launched at the end of May and data collection is projected to end in July 2024.

## Discussion

### Principal Findings

This protocol describes the research design and intended methodology for an eDelphi study to build consensus around the definition and risk stratification of immunosuppression in adults in the context of COVID-19. This work is a response to urgent calls to improve the precision of immunosuppressed disease surveillance [9]—something that is impossible without first establishing a universally accepted, clinically meaningful, and health record–compatible means of subdividing this diverse risk group. If fully executed, this protocol will achieve just this.

This study will be unique in its ability for panelists to leverage authors’ literature reviews and real-world evidence for differential immunosuppressed COVID-19 outcomes as preread materials. Likewise, the research group conducting this work has a global network of collaborators to call upon as panelists, including national and international health agencies and their respective vaccine advisory groups. Given the global implications of this work, we intend to secure a cross-continental panel with the highest possible caliber of panelists.

However, we anticipate that it will be difficult to achieve consensus on all questionnaire items at the level specified. The sheer scale and complexity of immunosuppression as a clinical risk group invites debate and likely ongoing disagreement. We predict that there may be discrepancies between the risks reported by condition-specific and condition-general panelists, for example. Condition-specific experts may report disproportionate vulnerability among their own patients. Likewise, geographical differences between panelists are likely to affect consensus. Those from less economically developed contexts may report elevated risks than those from contexts where medical provision is more assured. That said, there is a great advantage to capturing ongoing areas of dissent between international experts. Doing so will only improve the rigor of this investigation and the nuance of study insights and outputs that result.

## Appendix

Multimedia Appendix 1 Definition of immunosuppression in adult populations as per Green Book chapter 14a criteria.

## Abbreviations

DESTINIES: Delphi Study to Define and Risk-Stratify Immunosuppression
eDelphi: electronic Delphi
