# Supplementary material for: Defining and Risk-Stratifying Immunosuppression (the DESTINIES Study): Protocol for an Electronic Delphi Study
Source: JMIR Res Protoc. 2024 Jun 6;13:e56271. doi: 10.2196/56271 (PMC11190617; doi:10.2196/56271)

## Appendix 1:

## **Definition of immunosuppression in adult populations as per Green Book Chapter 14a Criteria**


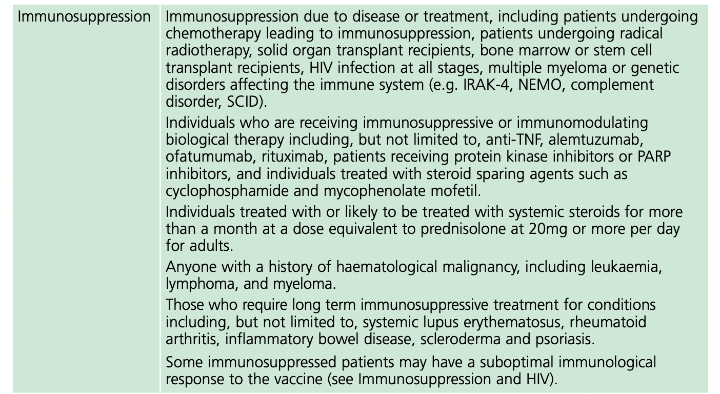

Supplement: Multimedia Appendix 1 [file resprot_v13i1e56271_app1.docx]
